# Interpretable machine learning model based on multimodal MRI radiomics for Alzheimer's disease diagnosis

**DOI:** 10.3389/fnagi.2026.1876233

**Published:** 2026-08-31

**Authors:** Nuerbiya Keranmu, Dilireba Aizezi, Xingyong Pan, Longtao Yang, Ying Liu, Jun Liu

**Affiliations:** 1 Department of Radiology, The Second Affiliated Hospital of Xinjiang Medical University, Ürümqi, China; 2 Department of Radiology, The Second Xiangya Hospital of Central South University, Changsha, Hunan, China

**Keywords:** Alzheimer's diagnosis, Alzheimer's disease, machine learning, magnetic resonance imaging, Shapley additive explanations

## Abstract

**Objective:**

Alzheimer's disease (AD), the most common neurodegenerative disorder, is a leading cause of cognitive impairment and dementia in older adults. This study aimed to develop an interpretable machine learning model using multimodal MRI radiomics for the diagnosis of Alzheimer's disease.

**Materials and methods:**

A total of 110 subjects (48 AD, 62 healthy control subjects) underwent 3D T1WI, DWI, and T2WI scans. Radiomics features were extracted from eight AD-related brain regions and selected using a three-step approach: variance thresholding, independent *t*-test, and LASSO–all conducted strictly within the training cohort. Logistic regression (LR) and random forest (RF) models were constructed for single-sequence and combined-sequence data. Model performance was evaluated using ROC analysis, calibration curves, and decision curve analysis. SHapley Additive exPlanations (SHAP) was applied for interpretability.

**Results:**

Sixteen core radiomics features were retained. Combined-sequence models outperformed single-sequence models, achieving test AUCs of 0.989 and 0.970 for LR and RF, respectively. The LR combined-sequence model achieved an accuracy of 0.882, sensitivity of 0.800, and specificity of 0.947. SHAP analysis identified texture features from the parietal lobe as key contributors. A nomogram integrating radiomics and clinical factors (homocysteine, triglycerides) demonstrated excellent calibration and clinical net benefit.

**Conclusion:**

Multimodal MRI radiomics combined with interpretable machine learning provides an accurate and explainable tool for AD diagnosis, with the combined LR model exhibiting superior performance.

## 1 Introduction

Alzheimer's disease (AD), the most common neurodegenerative disorder, is a leading cause of cognitive impairment and dementia in older adults. Its prevalence has been steadily increasing with the aging global population (Wang et al., 2019; Xu et al., 2023). The core pathological hallmarks of AD are extracellular deposition of β-amyloid (Aβ) plaques and intracellular accumulation of hyperphosphorylated tau protein forming neurofibrillary tangles (Sd'Errico and Meyer-Luehmann, 2020; Yarns et al., 2022). These changes begin years or even decades before clinical symptoms emerge. Early and accurate diagnosis of AD is therefore critical for delaying disease progression and improving patient outcomes (Rasmussen and Langerman, 2019).

Currently, clinical diagnosis of AD primarily relies on neuropsychological assessments, such as the Mini-Mental State Examination (MMSE), cerebrospinal fluid (CSF) biomarker analysis, and positron emission tomography (PET) (Milà-Alomà et al., 2019; Palmqvist et al., 2015). However, cognitive scales like the MMSE are influenced by the assessor's subjectivity and the subject's educational background, limiting their sensitivity in detecting early-stage mild cognitive impairment (MCI) (Yang et al., 2025; Jin and Wang, 2025). Although CSF and PET can accurately reflect the pathological status of Aβ and tau proteins, their invasive nature, high cost, and radiation exposure restrict their feasibility for large-scale screening and routine monitoring (Basanta-Torres et al., 2025; Wang et al., 2025). Structural magnetic resonance imaging (sMRI), a non-invasive, cost-effective, and widely accessible modality, can detect late-stage structural changes, such as hippocampal atrophy, but is less effective in identifying subtle early pathological alterations in AD (Gu et al., 2025; Rezaei et al., 2025; Beheshti et al., 2025). These limitations highlight the urgent need for a more efficient, non-invasive, and objective tool for early diagnosis.

Radiomics, an emerging medical image analysis technique, enables high-throughput extraction of quantitative features from medical images that are imperceptible to the human eye, facilitating deeper exploration of underlying pathophysiological information (Lambin et al., 2017; van Timmeren et al., 2020). In recent years, MRI radiomics combined with machine learning algorithms has shown considerable potential in AD research (Shi et al., 2024; Kale et al., 2024; Winchester et al., 2023). However, previous studies have faced two major limitations. First, most investigations have relied on a single MRI sequence, such as T1-weighted imaging, neglecting the complementary information provided by other sequences. For example, diffusion-weighted imaging (DWI) reflects microstructural integrity, while T2-weighted imaging is sensitive to lesion changes such as white matter hyperintensities. Multimodal MRI integration could therefore offer a more comprehensive depiction of AD pathology (Risacher and Saykin, 2013; Dang et al., 2023). Second, many high-performance machine learning models, particularly deep learning approaches, operate as “black boxes” with opaque decision-making processes, limiting interpretability and hindering clinical trust and translation (Ye et al., 2024; Wei et al., 2024; van der Velden et al., 2022).

To address these challenges, this study proposes a novel paradigm for AD diagnosis that integrates multimodal MRI with interpretable machine learning. Radiomics features and clinical information from three MRI sequences−3D-T1WI, DWI, and T2WI–were combined to construct multidimensional binary classification models and a nomogram using logistic regression (LR) and random forest (RF) algorithms. The LASSO algorithm was employed to identify the most diagnostically informative core feature combinations. In addition, SHapley Additive exPlanations (SHAP) analysis was applied to elucidate the internal decision-making process of the models and quantify the contribution of key features to diagnostic outcomes. This approach aims to provide an efficient, reliable, and interpretable imaging-assisted tool for precise clinical diagnosis of AD, thereby supporting early intervention and personalized treatment strategies.

## 2 Materials and methods

### 2.1 Study design and population

This retrospective cohort study included 110 subjects who either visited our hospital or underwent health examinations between January 2020 and November 2025. The cohort comprised 48 patients with AD aged 65 years or older and 62 age- and sex-matched healthy control (HC) subjects with comparable baseline characteristics, including living environment.

Inclusion criteria for AD patients:

① Fulfillment of the diagnostic criteria for AD according to the Chinese Guidelines for the Diagnosis and Treatment of AD (2021 edition), confirmed through comprehensive clinical history assessment, neuropsychological evaluation ((MMSE) score ≤ 26), and imaging examinations;
② Availability of complete head MRI scans, including 3D-T1WI, DWI, and T2WI sequences, with no significant artifacts (e.g., motion or metal artifacts) and clear visualization of the target brain regions;
③ Availability of complete clinical data (e.g., age, sex, disease duration), absence of severe systemic diseases (e.g., cardiac, hepatic, or renal disorders), no history of other central nervous system diseases (such as cerebrovascular disease, Parkinson's disease, or brain tumors), and no contraindications to MRI examination.

Exclusion criteria for the AD group:

① Presence of other neurological disorders that may cause cognitive decline, such as Parkinson's disease, intracranial tumors, vascular dementia, hydrocephalus, or dementia with Lewy bodies;
② Systemic diseases associated with cognitive impairment, including alcoholic encephalopathy, HIV infection, neurosyphilis, hypothyroidism, or vitamin B12 deficiency;
③ Drug- or toxin-induced dementia, such as from benzodiazepines, barbiturates, or antipsychotic medications;
④ Patients in a state of delirium;
⑤ Cognitive impairment secondary to psychiatric disorders, including major depression or schizophrenia;
⑥ History of malignant tumors, brain trauma, or cerebral hemorrhage;
⑦ Recent history of acute infection;
⑧ Hematological diseases or active/unknown bleeding sites;
⑨ Long-term use or use within the preceding 3 months of non-steroidal anti-inflammatory drugs (NSAIDs).

Inclusion criteria for the HC group:

① Cognitive function confirmed as normal through neuropsychological assessment (MMSE score ≥ 27);
② No significant structural abnormalities on head MRI and no family history of AD or other neurodegenerative diseases;
③ Fulfillment of all other inclusion criteria applied to the AD group.

Exclusion criteria for the HC group:

① Presence of conditions that may affect brain function, including definite personality disorders, psychiatric disorders, mood disorders, or migraines;
② Severe systemic disease, such as liver or kidney disease, diabetes, or other diseases affecting the nervous system;
③ History of long-term alcohol abuse or substance misuse;
④ Inability to adequately cooperate with or complete the examinations.

To ensure randomness and minimize selection bias, a stratified random sampling approach was adopted. This method preserved the proportion of AD cases in the training and test sets to reflect their distribution in the overall dataset. All subjects were randomly allocated to the training and test sets in a 7:3 ratio. During randomization, participants were first stratified according to key categorical variables, and a 7:3 split was then performed independently within each stratum. This procedure ensured comparable distributions of critical variables across the two datasets. As a result, the training set included 76 subjects (33 AD, 43 HC), and the test set included 34 subjects (15 AD, 19 HC). The two sets were comparable in terms of age (training set: 68.5 ± 7.2 years; test set: 69.3 ± 6.8 years) and sex distribution (training set, male/female: 39/37; test set, male/female: 18/16). The pooled cohort (all 110 subjects) had an overall mean age of 71.66 ± 7.2 years, with HC: 71.63 ± 6.8 years and AD: 71.69 ± 7.4 years (Table 1). This study was approved by the Medical Ethics Committee of our hospital (Approval No.: KY2025010906) and conducted in accordance with the Declaration of Helsinki. Due to its retrospective design, the requirement for informed consent was waived.

**Table 1:** Demographic characteristics of the healthy control group and Alzheimer's disease patients group characteristic.

Characteristic	AD *n* = 48	HC *n* = 62	Statistical method	Test statistic	*p*-value
Age (years)	71.69 ± 7.4	71.63 ± 6.8	*t*-test	*t* = 0.043	0.966
Gender (Male/Female)	21/27	28/34	Chi-square	χ^2^ = 0.021	0.884
BMI *kg*/*m*2	23.5 ± 3.2	24.1 ± 2.9	*t*-test	*t* = 0.842	0.402
MMSE score	17.3 ± 4.1	28.7 ± 1.1	*t*-test	*t* = 15.237	< 0.001

Data are mean ± SD or *n* (%). Training and test set sub-distributions are detailed in Section 2.1. BMI, body mass index; MMSE, Mini-Mental State Examination.

### 2.2 Imaging protocol and parameters

All participants underwent head MRI scans on a 3.0T MR scanner (Philips) at our institution, using a 32-channel head coil. Participants were positioned supine, with their heads immobilized to minimize motion artifacts. The scanning sequences and parameters were as follows:

3D-T1WI (thin-slice T1-weighted imaging):Acquired using a three-dimensional magnetization-prepared rapid gradient echo (3D-MPRAGE) sequence. Parameters were: repetition time (TR) = 7.9 ms; echo time (TE) = 3.6 ms; flip angle = 90°; field of view (FOV) = 240 mm × 240 mm; matrix size = 220 × 219; slice thickness = 1 mm with no interslice gap; total acquisition time = 5 min 8 s. This sequence was primarily used to provide high-resolution delineation of gray-white matter boundaries, serving as the anatomical reference for brain region segmentation.

DWI Sequence: a single-shot echo-planar imaging (EPI) sequence was employed with the following parameters: TR = 2,983 ms; TE = 81 ms; FOV = 230 mm × 230 mm; matrix size = 164 × 122; slice thickness = 5 mm; interslice gap = 1 mm; b-values = 0 and 1,000 s/mm^2^; diffusion gradient directions = 6; total acquisition time = 36 s. This sequence measures the diffusion of water molecules in brain tissue, providing insights into microstructural alterations.

T2 Sequence (T2-weighted imaging):A fast spin-echo sequence was employed with parameters: TR = 3,000 ms; TE = 80 ms; FOV = 230 mm × 182 mm; matrix size = 420 × 264; slice thickness = 5 mm; interslice gap = 1 mm; total acquisition time = 1 min 12 s. This sequence is sensitive to brain tissue edema, inflammation, and early pathological changes, thereby facilitating the detection of abnormal signal alterations in AD-related brain regions.

All original images were exported from the hospital's picture archiving and communication system (PACS) in DICOM format for subsequent brain region segmentation and radiomics feature extraction.

### 2.3 Region of interest (ROI) segmentation

First, spatial registration of the three MRI sequences (3D-T1WI, DWI, and T2WI) was performed using ANTs software. The 3D-T1WI sequence was designated as the fixed image, while the DWI and T2WI sequences served as moving images. The registration procedure consisted of: bias field correction using the N4BiasFieldCorrection module; skull stripping using the ExtractBrainMask module; and linear plus nonlinear registration using the Symmetric Normalization (SyN) algorithm to achieve precise spatial alignment between the moving and fixed images.

Subsequently, fully automated whole-brain segmentation was conducted on the registered 3D-T1WI images using FastSurfer (https://github.com/Deep-MI/FastSurfer). This framework employs a deep learning model trained on a large-scale structural brain database of healthy individuals and enables accurate segmentation of 95 anatomical brain regions, including detailed parenchymal extraction and regional labeling. It also provides reliable segmentation of AD-related regions, such as the hippocampus and entorhinal cortex. Based on established pathological mechanisms of AD and relevant clinical evidence, eight core brain regions were selected from the 95 segmented regions: the hippocampus, temporal lobe, frontal lobe, occipital lobe, parietal lobe, thalamus, entorhinal cortex, and posterior cingulate cortex.

Finally, the segmentation results were reviewed under blinded conditions by a chief physician with 10 years of experience in neuroimaging diagnosis to ensure that the boundaries of the eight target brain regions corresponded accurately to anatomical structures. For regions with ambiguous boundaries due to atrophy, such as the entorhinal cortex and hippocampus, manual corrections were performed using ITK-SNAP software. The following standardized anatomical criteria were applied for manual adjustments: the hippocampus was delineated based on the characteristic “seahorse” shape on coronal sections, with boundaries defined by the temporal horn of the lateral ventricle, the parahippocampal gyrus, and the choroidal fissure; the entorhinal cortex was defined on coronal sections using the rhinal sulcus as the lateral border and the uncus as the medial reference (Insausti et al., 1998). Although formal inter-observer variability metrics, such as Dice similarity coefficients, were not systematically calculated–which the authors acknowledge as a limitation–all adjustments were performed by a single experienced radiologist with standardized criteria to ensure internal consistency. Future studies will implement dual-operator segmentation with inter-observer reliability assessment to enhance reproducibility. This process ensured that the ROIs fully encompassed the target brain areas while excluding irrelevant structures, resulting in a standardized ROI dataset for subsequent multi-sequence radiomics feature extraction.

### 2.4 Radiomics feature extraction and selection

Radiomics features were extracted from standardized ROIs of eight core brain regions using the PyRadiomics toolkit (version 3.0.1, https://pyradiomics.readthedocs.io/). Features were obtained separately for each brain region in each subject, resulting in a multi-dimensional feature dataset. In total, 856 features were extracted, with 107 features per brain region, covering three main categories:

First-order statistical features: including mean, variance, skewness, kurtosis, total energy, 10th percentile, 90th percentile, interquartile range, and others, reflecting the overall distribution of voxel intensities within the ROI.

Shape features: such as major axis length, volume, and sphericity, quantifying the geometric morphology and spatial size of the ROI.

Texture features: derived from gray level co-occurrence matrix (GLCM), gray level run length matrix (GLRLM, e.g., short run high gray level emphasis), gray level size zone matrix (GLSZM, e.g., gray level non-uniformity), gray level dependence matrix (GLDM, e.g., small dependence emphasis), and neighboring gray tone difference matrix (NGTDM, e.g., contrast), capturing the heterogeneity and spatial distribution patterns of gray-level intensities within the ROI.

A three-step feature selection procedure was employed to remove redundant information and retain key diagnostic features. All selection procedures were conducted exclusively within the training cohort *n* = 76, with the test cohort *n* = 34 completely held out and used solely for final model evaluation. First, a variance threshold method was applied with a threshold of 0.8. The variance of all features was calculated within the training set, and features with low variability (variance < 0.8) were removed, thereby preliminarily eliminating redundant information with minimal discriminative value. Second, univariate feature selection was conducted. For the standardized features within the training set, independent samples *t*-tests were used to assess differences between the AD and HC groups (for continuous variables). Features with *p*-values ≥ 0.05 were considered not significantly associated and were excluded. Third, feature screening was performed using the LASSO (Least Absolute Shrinkage and Selection Operator) algorithm within the training set. The optimal regularization parameter was determined via 5-fold cross-validation using only training data. By applying an L1 regularization penalty, redundant feature coefficients were shrunk to zero. This three-step process ultimately yielded a set of core radiomics features for subsequent model construction.

### 2.5 Model construction and interpretability analysis

Based on the selected core radiomics features, eight binary classification models for AD were constructed using two classical machine learning algorithms, LR and RF, applied to three individual MRI sequences (3D-T1WI, DWI, T2) as well as their combination (Combined). The specific models were: 3D-T1WI-LR, 3D-T1WI-RF, DWI-LR, DWI-RF, T2-LR, T2-RF, Combined-LR, and Combined-RF. Model training and validation were conducted in Python 3.9, using the scikit-learn 1.3.0 library for algorithm implementation. In addition, univariate and multivariate analyses were performed to identify key clinical factors from the collected patient data. Based on these factors, a clinical model (Clic-LR and Clic-RF) and a nomogram model combining radiomics and clinical features (Nomogram model) were developed.

Model evaluation followed a “training set training–test set validation” workflow. Specifically, 76 samples from the training set were used for model parameter optimization and training, while the remaining 34 samples from the test set were employed to assess the model's generalization ability. Diagnostic performance was evaluated using the following metrics: (1) receiver operating characteristic (ROC) curve and area under the curve (AUC): quantifying the model's overall ability to discriminate between AD and HC. (2) Confusion matrix: from which accuracy (ACC), sensitivity (SEN), and specificity (SPE) were calculated: ACC = number of correctly predicted samples / total number of samples; SEN = true positives/(true positives + false negatives), reflecting the model's ability to identify AD patients; SPE = true negatives/(true negatives + false positives), reflecting the model's ability to identify HC individuals. Additionally, a nomogram was plotted for the nomogram-based model, and both clinical benefit analysis and calibration analysis were conducted to further evaluate its predictive performance.

To elucidate the decision-making mechanism of the model, SHAP interpretability analysis was applied to the best-performing Combined-LR model. This analysis produced several visualizations: a heatmap showing the sample-feature SHAP value distribution and indicating whether features promoted or inhibited the predicted outcome; a beeswarm plot illustrating the ranking of feature importance and the relationship between feature values and SHAP values; a decision plot depicting the formation of the predicted probability for individual samples; and waterfall charts breaking down the contribution of each feature for a single sample.

Statistical analyses were conducted using R (version 4.4.0) and Python (version 3.9). Model evaluation results are reported as mean values with 95% confidence intervals. Final outputs included a performance evaluation table (reporting AUC, accuracy, sensitivity, and specificity for both training and test sets), along with ROC curves, confusion matrices, and SHAP plots to visually convey model performance.

## 3 Results

### 3.1 Demographic characteristics

A total of 110 subjects were included in the analysis, consisting of 62 HC and 48 patients with AD. Of these participants, 49 were male and 61 were female, with an age range of 66–87 years (mean ± standard deviation). The demographic characteristics of the two groups are summarized in Table 1. No significant differences in age or gender were observed between the groups.

Univariate and multivariate analyses revealed that homocysteine and triglyceride levels differed significantly between the AD and HC groups (*p* < 0.05), as shown in Table 2.

**Table 2:** Comparison of clinical laboratory parameters between AD and HC groups characteristic.

Characteristic	AD *n* = 48	HC *n* = 62	Statistical method	Test statistic	*p*-value
Homocysteine μ*mol*/*L*	15.6 ± 3.8	11.2 ± 2.9	*t*-test	*t* = 5.872	< 0.001
Triglycerides (mmol/L)	2.1 ± 0.9	1.5 ± 0.6	*t*-test	*t* = 4.103	< 0.001
Total cholesterol (mmol/L)	5.1 ± 1.2	5.0 ± 1.1	*t*-test	*t* = 0.445	0.657
HDL (mmol/L)	1.2 ± 0.3	1.3 ± 0.3	*t*-test	*t* = 1.674	0.097
LDL (mmol/L)	3.0 ± 0.9	2.9 ± 0.8	*t*-test	*t* = 0.602	0.549
Glucose (mmol/L)	5.4 ± 0.8	5.2 ± 0.7	*t*-test	*t* = 1.384	0.169
Hypertension (*n*, %)	22 (45.8%)	25 (40.3%)	Chi-square	χ^2^ = 0.342	0.559
Diabetes (*n*, %)	10 (20.8%)	12 (19.4%)	Chi-square	χ^2^ = 0.037	0.847

Data are mean ± SD or *n* (%). Training and test set sub-distributions are detailed in Section 2.1. BMI, body mass index; MMSE, mini-mental state examination.

### 3.2 Results of radiomics feature selection

**Figure 1:**
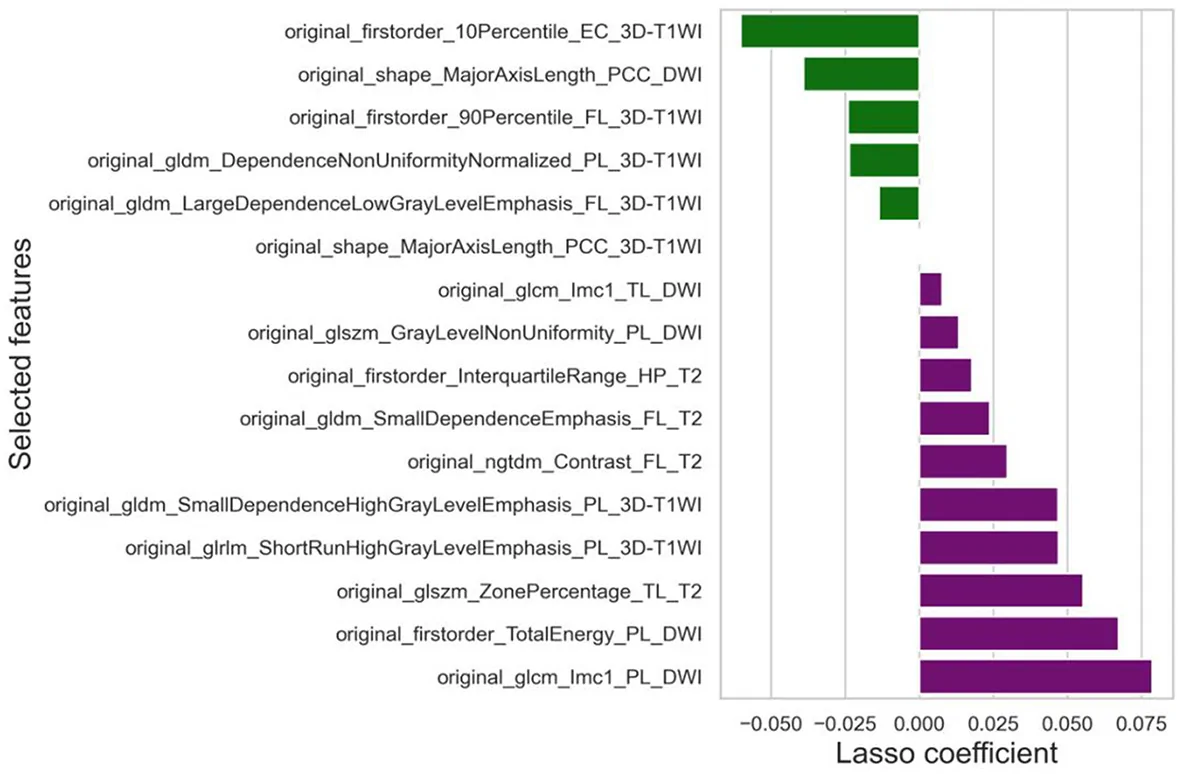
Final radiomics features selected by the combined model.

In this study, a total of 856 radiomics features were initially extracted from eight core brain region ROIs. Following the application of a three-step feature selection procedure, 16 core features were ultimately retained. The selected features and their corresponding coefficients are presented in Figure 1 and summarized in Table 3. Among these 16 features, 10 were texture features, four were first-order statistical features, and two were shape features. Texture features derived predominantly from the GLCM and GLRLM, which together accounted for seven features, representing 70.0% of all texture features. With respect to their anatomical distribution, the parietal lobe contributed the greatest number of features *n* = 5, followed by the frontal lobe *n* = 3, posterior cingulate cortex *n* = 2, temporal lobe *n* = 2, hippocampus *n* = 2, and entorhinal cortex *n* = 2. No core features were selected from the thalamus or occipital lobe. In terms of imaging sequence distribution, six features were derived from the 3D-T1WI sequence, five from the DWI sequence, and five from the T2WI sequence, indicating a relatively balanced contribution across the three modalities.

**Table 3:** The 16 radiomics features selected by the combined model.

Radiomic features	Coefficient
original_glcm_Imc1_PL_DWI	0.07859
original_glrlm_ShortRunHighGrayLevelEmphasis_PL_3D-T1WI	0.04705
original_glszm_GrayLevelNonUniformity_PL_DWI	0.01338
original_gldm_SmallDependenceEmphasis_FL_T2	0.02388
original_firstorder_TotalEnergy_PL_DWI	0.06735
original_glcm_Imc1_TL_DWI	0.00772
original_shape_MajorAxisLength_PCC_DWI	−0.03896
original_ngtdm_Contrast_FL_T2	0.02974
original_gldm_DependenceNonUniformityNormalized_PL_3D-T1WI	−0.02358
original_shape_MajorAxisLength_PCC_3D-T1WI	−0.00056
original_glszm_ZonePercentage_TL_T2	0.05528
original_firstorder_90Percentile_FL_3D-T1WI	−0.02401
original_gldm_LargeDependenceLowGrayLevelEmphasis_FL_3D-T1WI	−0.01353
original_firstorder_10Percentile_EC_3D-T1WI	−0.06018
original_gldm_SmallDependenceHighGrayLevelEmphasis_PL_3D-T1WI	0.04685
original_firstorder_InterquartileRange_HP_T2	0.01775

Among the selected core features, notable changes included an increase in the GLRLM feature from the parietal lobe in the 3D-T1WI sequence (original_glrlm_ShortRunHighGrayLevelEmphasis_PL_3D-T1WI), an increase in the GLCM feature from the parietal lobe in the DWI sequence (*original*_*g*_*lcm*_*I*_*mc*1_*P*_*L*_*D*_*WI*), and a decrease in the shape feature from the posterior cingulate cortex in the DWI sequence (*original*_*s*_*hape*_*M*_*ajorAxisLength*_*P*_*CC*_*D*_*WI*).

### 3.3 Model evaluation

**Figure 2:**
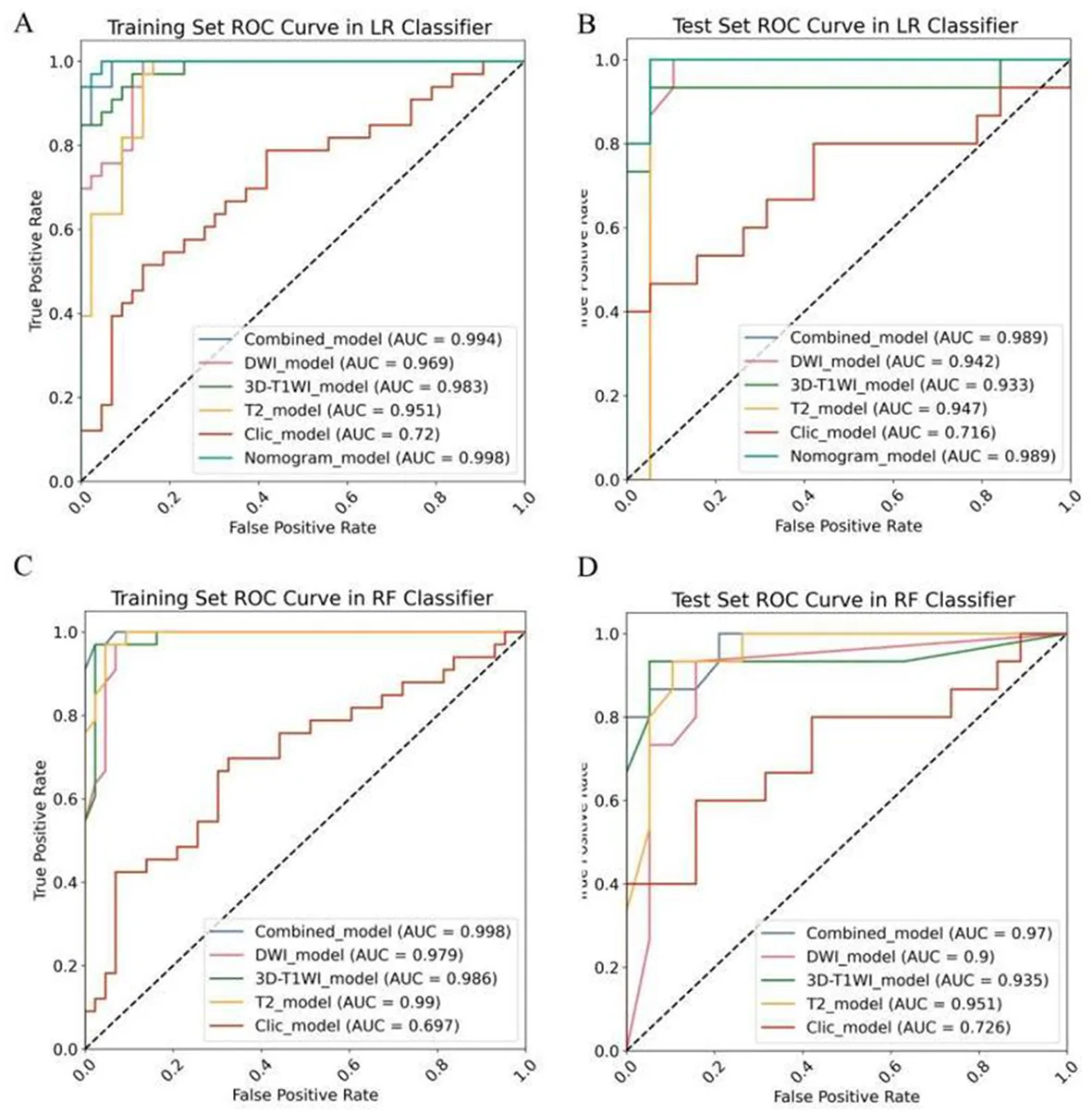
ROC curves for all models. **(A, B)** ROC curves for all LR models; **(C, D)**, ROC curves for all RF models.

All radiomics-based diagnostic models for AD developed in this study demonstrated strong performance in both the training and test sets. The evaluation metrics, including the AUC, accuracy, sensitivity, and specificity, are summarized in Table 3. The ROC curves for all models are presented in Figure 2.

In the training set, all radiomics models achieved AUC values exceeding 0.950, with the combined-sequence models demonstrating the best performance. The combined-LR and combined-RF models attained AUCs of 0.994 (95% CI: 0.983–1.000) and 0.998 (95% CI: 0.992–1.000), respectively, both with an accuracy of 0.961. The combined-LR model achieved perfect sensitivity (1.000), indicating no missed diagnoses, while the combined-RF model achieved the highest specificity (0.953). Among single-sequence models, those based on 3D-T1WI and T2 sequences performed better, with the 3D-T1WI-RF and T2-RF models achieving training set AUCs of 0.986 (95% CI: 0.965–1.000) and 0.990 (95% CI: 0.975–1.000), respectively. In the test set, the combined-sequence models maintained superior performance. The Combined-LR model achieved an AUC of 0.989 (95% CI: 0.960–1.000), with an accuracy of 0.882 and a specificity of 0.947. The Combined-RF model reached an AUC of 0.970 (95% CI: 0.920–1.000), with an accuracy of 0.912 and sensitivity of 0.867; both models shared the same specificity. Among single-sequence models, the T2-LR model showed outstanding test-set performance, with an AUC of 0.947 (95% CI: 0.850–1.000), accuracy of 0.971, and perfect sensitivity (1.000). The 3D-T1WI-based models (3D-T1WI-LR and 3D-T1WI-RF) achieved a sensitivity of 0.933. In contrast, DWI-based models exhibited the lowest performance, with the DWI-RF model reaching a test-set AUC of only 0.900 (95% CI: 0.788–0.987) and a specificity of 0.842. By comparison, the clinical-only models showed substantially lower diagnostic performance. Among the LR and RF algorithms, the RF-based clinical model achieved the highest test-set AUC of just 0.726 (95% CI: 0.568–0.879), underscoring the superior predictive value of radiomic features over clinical variables alone.

**Figure 3:**
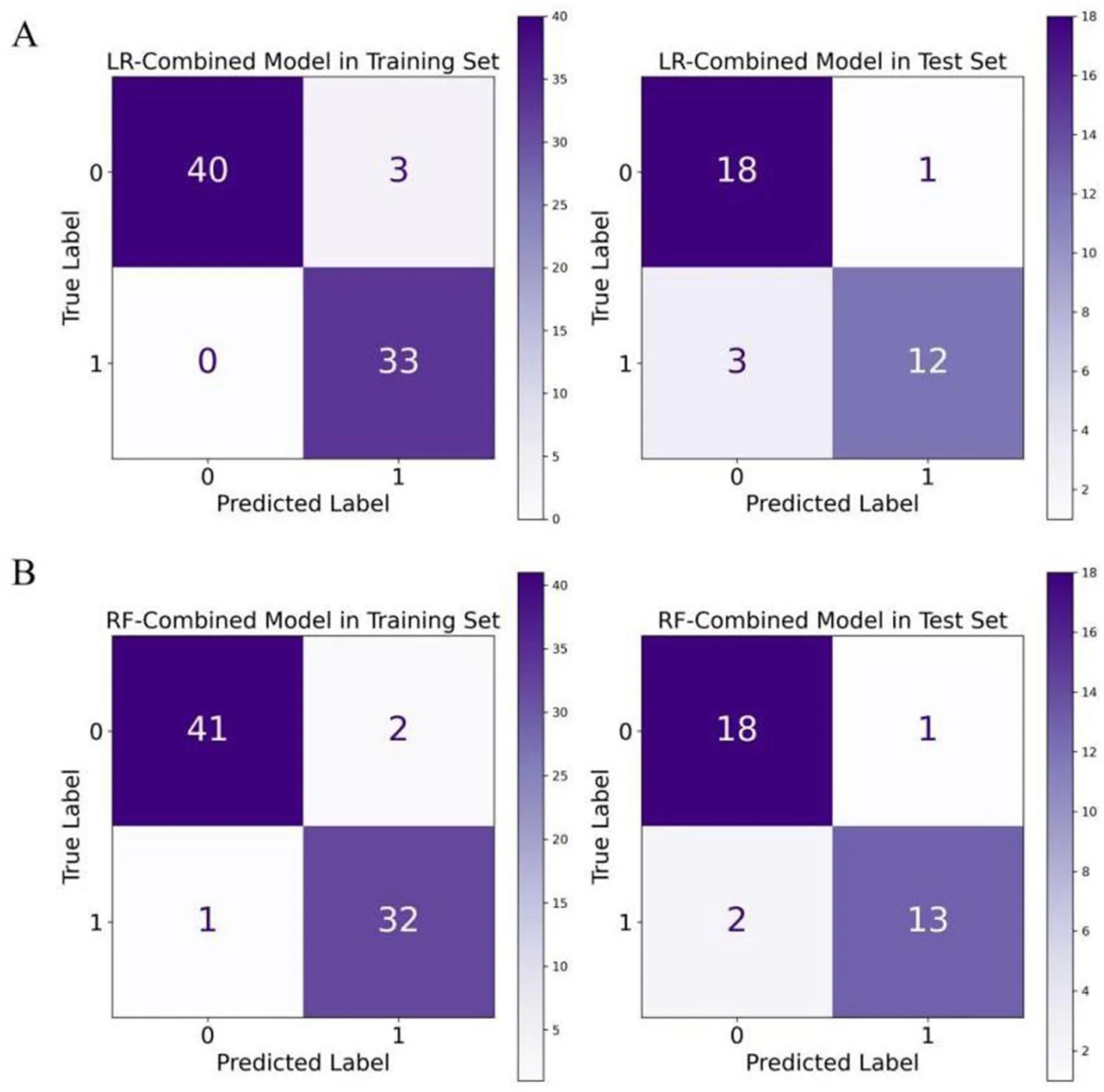
Shows the confusion matrices of the combined models. **(A)** confusion matrix plot of the combined model for LR; **(B)** confusion matrix plot of the combined model for RF.

As shown in Figure 3, the confusion matrix results for the combined models are as follows. In the training set, the combined-LR model correctly classified 40 of 43 HC and all 33 AD patients, with 3 HC samples misclassified as AD. The RF-Combined model correctly identified 41 HC and 32 AD cases, with 2 HC misclassified as AD and 1 AD case misclassified as HC. In the test set, the LR-Combined model correctly classified 18 of 19 HC and 12 of 15 AD cases, with one HC and three AD samples misclassified. The RF-Combined model achieved similar results, correctly classifying 18 HC and 13 AD cases, with one HC and two AD samples misclassified. Overall, both models demonstrated strong and comparable classification performance on the independent test set, reflecting robust generalizability and reliable predictive capability.

### 3.4 Interpretability analysis

**Figure 4:**
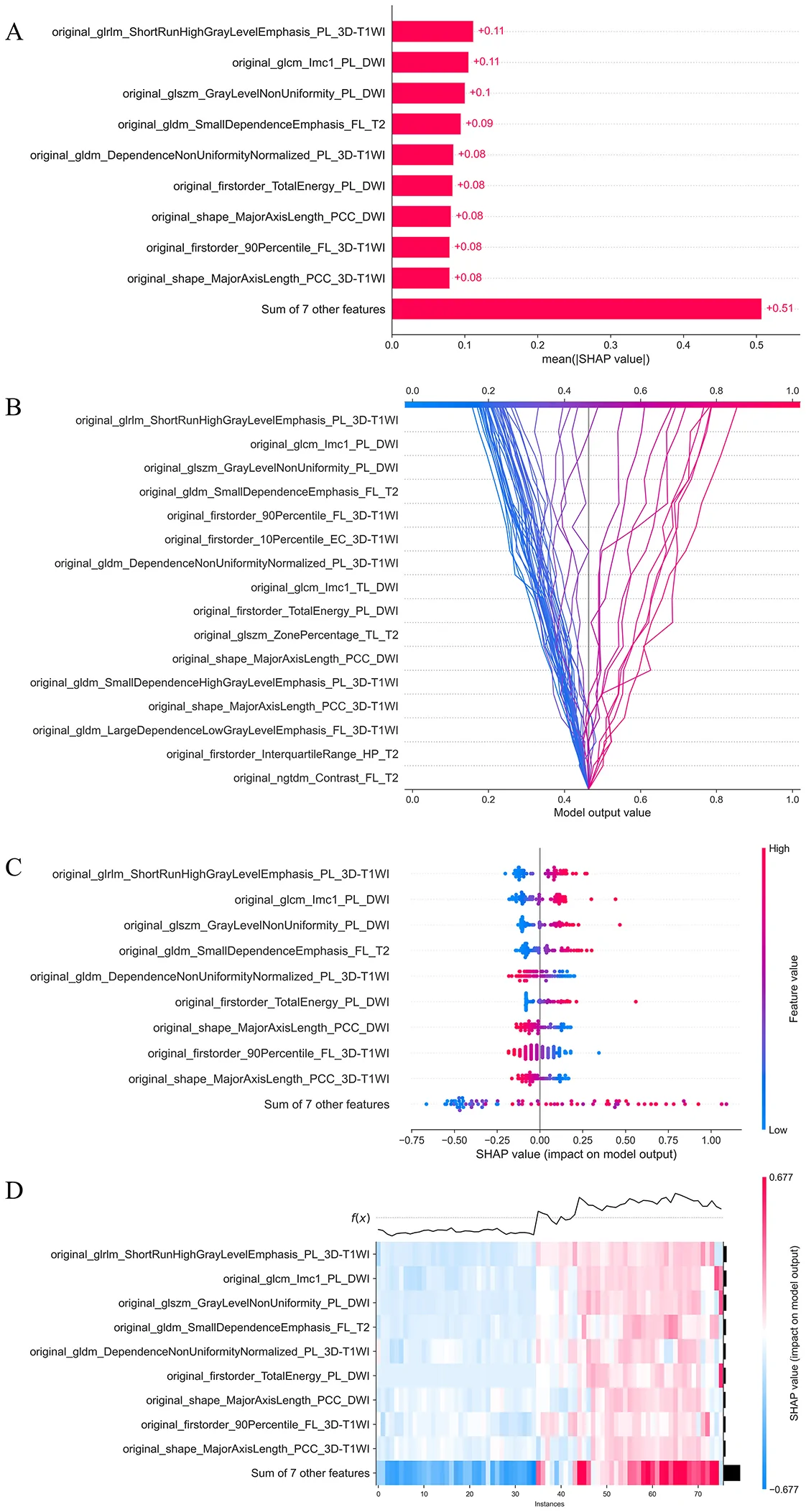
Results of SHAP analysis plots. **(A)** Feature weight bar plot; **(B)** SHAP decision plot for samples; **(C)** SHAP swarm plot; **(D)** SHAP heatmap for samples.

The interpretability analysis of the combined-LR model (Figure 4) revealed that texture features of the parietal lobe, specifically original_glrlm_ShortRunHighGrayLevelEmphasis_PL_3D-T1WI and original_glcm_Imc1_PL_DWI, serve as key positive contributors driving the model's predictions toward AD. In contrast, the 3D-T1WI-derived gray-level dependence matrix feature of the parietal lobe (original_gldm_DependenceNonUniformityNormalized_PL_3D-T1WI) acts as a core negative feature, suppressing AD prediction. Together, these three features form the principal basis for distinguishing AD from normal controls, with the directionality of their contributions aligning closely with known pathological mechanisms underlying microstructural abnormalities in the parietal lobe of AD patients.

Among the interpretability analyses, the SHAP bar chart (Figure 4A) presents each feature on the vertical axis and the mean absolute SHAP value on the horizontal axis, intuitively illustrating the overall contribution of each feature to the model's predictions. The results indicate that original_glrlm_ShortRunHighGrayLevelEmphasis_PL_3D-T1WI has the highest mean absolute SHAP value, followed by original_glcm_Imc1_PL_DWI and original_gldm_DependenceNonUniformityNormalized_PL_3D-T1WI; identifying these three as the core features driving the model's diagnostic decisions. The SHAP decision plot (Figure 4B) displays features along the vertical axis and the predicted probability of AD along the horizontal axis. Each curve traces the cumulative effect of feature SHAP values on the final prediction for a single sample, starting from the baseline probability of 0.5. For samples predicted as AD (probability > 0.5), the prediction is predominantly influenced by high SHAP values of the core positive features. Conversely, samples predicted as HC (probability < 0.5) are primarily driven by low SHAP values of the core negative features. Samples with predicted probabilities near 0.5, representing atypical or borderline cases, exhibit offsetting contributions due to the cancellation of positive and negative SHAP effects. The SHAP swarm plot (Figure 4C) further visualizes the relationship between feature values and their impact on predictions. Features are plotted on the vertical axis, with SHAP values on the horizontal axis, and each point represents a single sample. Feature values are color-coded (red for high, blue for low). The results show that high values of original_glrlm_ShortRunHighGrayLevelEmphasis_PL_3D-T1WI and original_glcm_Imc1_PL_DWI correspond to positive SHAP values, while high feature values of original_gldm_DependenceNonUniformityNormalized_PL_3D-T1WI correspond to negative SHAP values, clearly demonstrating the directionality of feature contributions. Finally, the SHAP heatmap (Figure 4D) depicts samples along the horizontal axis and features along the vertical axis, with color intensity reflecting SHAP value magnitude (red for positive, blue for negative). This visualization provides an intuitive overview of how individual feature contributions correspond to diagnostic labels across the dataset, further validating the critical role of the identified core features in the model's decision-making process.

### 3.5 Nomogram construction

**Figure 5:**
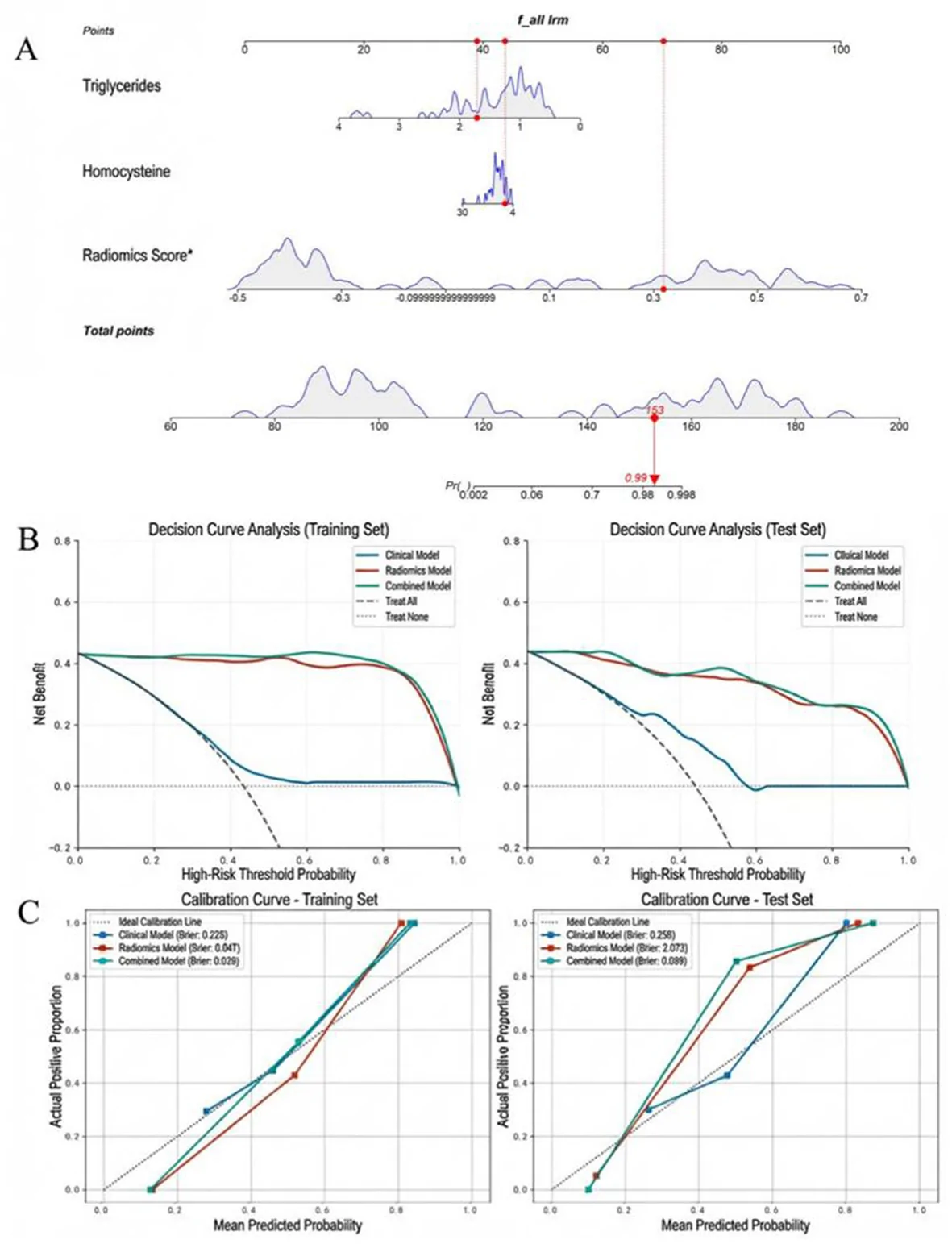
Curves related to the nomogram. **(A)** Nomogram; **(B)** Decision curve analysis curve; **(C)** Calibration curve.

Based on the selected core radiomic features and key clinical variables identified through univariate and multivariate analyses, particularly homocysteine and triglyceride levels, a combined radiomics–clinical nomogram was developed, and its graphical representation is shown in (Figure 5A). In the training cohort, the nomogram demonstrated excellent discriminatory performance, achieving an AUC of 0.998 (95% CI: 0.993–1.000), with an accuracy of 0.961, a sensitivity of 1.000, and a specificity of 0.930. In the independent test cohort, the model maintained robust performance, yielding an AUC of 0.989 (95% CI: 0.960–1.000), consistent with that of the Combined-LR model. The corresponding accuracy, sensitivity, and specificity were 0.912, 0.800, and 1.000, respectively, indicating strong diagnostic stability and generalizability. The calibration curves (Figure 5B) demonstrated excellent agreement between predicted probabilities and observed diagnostic outcomes, with low Brier scores of 0.039 in the training set and 0.059 in the test set, reflecting high predictive reliability. Furthermore, decision curve analysis (**DCA**, Figure 5C) showed that across a broad range of clinically relevant high-risk threshold probabilities, the nomogram provided a greater net benefit than the clinical-only model as well as the “treat-all” and “treat-none” strategies. These findings underscore the substantial clinical utility and practical applicability of the proposed radiomics–clinical nomogram.

## 4 Discussion

This study developed an interpretable machine learning model based on multimodal MRI radiomics and constructed a practical nomogram for the auxiliary diagnosis of AD. The results demonstrated that the combined model, integrating 3D-T1WI, DWI, and T2 sequences, achieved superior diagnostic performance, substantially outperforming any single-sequence model and exhibiting diagnostic efficacy comparable to that of the nomogram incorporating both radiomic and clinical factors. Furthermore, through SHAP analysis, this study elucidated the internal decision-making process of the previously “black-box” model and confirmed that the core imaging features identified correspond closely with established pathophysiological mechanisms of AD. Clinical factors, such as homocysteine and triglyceride levels, further enhanced specificity, highlighting the complementary value of integrating imaging and biochemical markers. The integration of multimodal MRI with interpretable machine learning represents a paradigm shift in AD imaging diagnosis, moving from purely morphological assessments toward a multiparametric, systems-biology approach. Specifically, this study addresses four critical challenges: comprehensive pathological profiling: by combining structural, functional, and microstructural sequence information, the model overcomes “information silos” and constructs a multidimensional representation of AD pathology. Early and precise diagnosis: the model identifies subtle multimodal patterns that are imperceptible to the naked eye, enabling early disease detection and precise diagnosis beyond conventional “*post-hoc*” assessments that rely on overt regional atrophy. Individualized disease characterization: the interpretable outputs reveal the heterogeneity of AD, providing a foundation for personalized subtyping and prognostic evaluation. Clinical translation: by transforming the predictive model into a practical nomogram, this study facilitates the implementation of artificial intelligence as a tangible decision-support tool in clinical settings. Overall, this framework bridges the gap between advanced AI-driven imaging analysis and real-world clinical application, offering a robust, interpretable, and clinically actionable approach to AD diagnosis and management.

### 4.1 Relationship between homocysteine, triglyceride metabolic abnormalities, and AD

Based on the selected core radiomic features and key clinical variables, including homocysteine and triglyceride levels identified through univariate and multivariate analyses, a combined radiomics–clinical nomogram was developed. This integrated model demonstrated excellent and consistent diagnostic performance in both the training and independent test sets, with robust accuracy, sensitivity, and specificity. These findings underscore its potential clinical applicability and translational value. Importantly, the results support the hypothesis that dysregulation of homocysteine and triglyceride metabolism constitutes a significant component of the interconnected pathophysiological network underlying AD. These metabolic abnormalities may synergistically accelerate disease progression through multiple overlapping mechanisms involving vascular injury, metabolic dysfunction, and neuroinflammatory pathways.

1. Vascular mechanisms. Elevated homocysteine levels can directly impair cerebrovascular integrity and disrupt the blood–brain barrier (BBB) by inducing endothelial dysfunction, oxidative stress, and inflammatory responses. Meanwhile, triglycerides, central contributors to atherosclerosis and cerebral microangiopathy, promote chronic hypoperfusion and ischemic injury. Emerging evidence suggests that these two factors may act additively or synergistically to aggravate cerebrovascular damage. BBB disruption may arise not only from the direct neurovascular toxicity of homocysteine but also from systemic vascular inflammation associated with hypertriglyceridemia. Together, these processes contribute to neuronal hypoxia, insufficient nutrient delivery, and impaired clearance of neurotoxic metabolites.
2. Metabolic dysfunction and insulin resistance. Homocysteine has been implicated in the exacerbation of insulin resistance, while insulin resistance itself disrupts lipid metabolism, forming a self-reinforcing pathological cycle. This interaction may impair central insulin signaling pathways, leading to neuronal glucose hypometabolism, mitochondrial dysfunction, and energy deficits. Furthermore, disrupted metabolic signaling may directly promote amyloid-β (Aβ) oligomerization and Tau hyperphosphorylation, thereby accelerating core AD pathological processes.
3. Neuroinflammation and oxidative stress. During its metabolism, homocysteine generates reactive oxygen species and activates microglia, triggering local neuroinflammatory cascades. Concurrently, pro-inflammatory cytokines, such as IL-6 and TNF-α, released from adipose tissue, particularly visceral fat, may cross a compromised BBB or influence the central nervous system via neurohumoral pathways. This external inflammatory influx collaborates with the localized inflammation induced by homocysteine, creating a synergistic effect that jointly promotes the pathological progression of AD.

### 4.2 Complementary value of multimodal MRI sequences in AD diagnosis

This study systematically evaluated the diagnostic performance of three individual MRI sequences and a multimodal combined-sequence model for AD. The findings demonstrated that the combined model significantly outperformed all single-sequence models, achieving test-set AUC values of 0.989 for the LR classifier and 0.970 for the RF classifier. These results underscore the clear advantage of multimodal information fusion in improving diagnostic accuracy. This observation is consistent with a growing body of literature suggesting that integrating complementary imaging modalities enables a more comprehensive characterization of the complex pathological landscape of AD, thereby enhancing predictive performance (Ismail et al., 2022; Tang et al., 2024). By fusing radiomic features derived from multiple sequences, the present framework effectively captures diverse yet interrelated aspects of disease pathology, forming a complementary and synergistic representation of AD-related alterations. Specifically, 3D-T1-weighted imaging (3D-T1WI), widely regarded as the reference standard for evaluating brain structural atrophy, provides precise quantification of macroscopic morphological changes in regions such as the hippocampus and cerebral cortex. DWI, through the assessment of water molecule diffusion properties, reveals microstructural abnormalities that may emerge during the early stages of AD, including neuronal injury and myelin disruption. In parallel, T2-weighted imaging demonstrates heightened sensitivity to early pathological responses, such as astrocytic proliferation, microvascular alterations, and tissue edema. Together, the integration of these sequences enables a multidimensional assessment of AD pathology, spanning macroscopic structural degeneration, microstructural integrity, and early reactive changes, thereby substantially strengthening the biological and diagnostic relevance of the model.

Among the single-sequence models, the T2-weighted model demonstrated the most stable and favorable diagnostic performance on the test set (AUC = 0.947 for the LR model and AUC = 0.951 for the RF model). This superiority may be attributed to the sensitivity of T2-weighted imaging in detecting early, non-specific pathological alterations, such as neuroinflammation and tissue edema, which may precede overt macroscopic structural atrophy. In contrast, the DWI-based model exhibited comparatively lower diagnostic performance. This may be explained by the subtlety of water diffusion abnormalities during the early stages of AD, as well as the susceptibility of diffusion-derived quantitative metrics to variations in acquisition parameters (e.g., b-value selection). These factors may limit the robustness of DWI features when used in isolation for diagnostic purposes. The successful development of the multimodal model effectively integrates complementary pathological information across sequences: macroscopic structural characteristics from 3D-T1WI, microstructural integrity markers from DWI, and early pathological alterations captured by T2-weighted imaging. By achieving multidimensional pathological coverage, spanning both structural and functional domains, as well as macroscopic and microscopic levels, the combined framework overcomes the informational constraints inherent in single-sequence models and delivers more stable and accurate diagnostic performance. Importantly, this multimodal strategy does not represent a simple aggregation of features. Rather, the applied algorithms learn complex and potentially nonlinear interactions among radiomic features derived from different sequences, thereby constructing a more comprehensive and biologically informed pathobiological representation of AD.

### 4.3 Machine learning algorithm selection and model performance comparison

To evaluate the adaptability of different algorithms in modeling high-dimensional radiomic data, this study compared a linear LR classifier with a nonlinear RF ensemble model. The results demonstrated that the combined multimodal models constructed using both algorithms achieved excellent diagnostic performance. Specifically, the Combined-LR model attained a higher AUC on the independent test set (0.989 vs. 0.970) and exhibited strong specificity (0.947). This finding highlights its advantage in accurately distinguishing HC and minimizing false-positive classifications, which is particularly important for reducing unnecessary medical interventions and associated healthcare burdens. In contrast, the Combined-RF model showed slightly superior overall accuracy (0.912 vs. 0.882) and sensitivity (0.867 vs. 0.800), indicating a greater capacity to correctly identify patients with AD and to reduce the risk of missed diagnoses. These characteristics suggest that the RF-based model may be particularly valuable in clinical contexts where early detection and case identification are prioritized.

The observed performance differences between the two algorithms can be attributed to their distinct underlying mechanisms. LR, characterized by its relatively simple structure and computational efficiency, directly models the linear contributions of individual imaging features through estimated feature coefficients. Its strong performance in the present study suggests that the selected core radiomic features exhibit substantial linear separability with respect to AD diagnosis (Watanabe et al., 2021). In contrast, RF, as an ensemble learning approach, constructs multiple decision trees and aggregates their predictions to generate a final output. This architecture enables RF to effectively capture nonlinear relationships and complex feature interactions, rendering it particularly well suited for heterogeneous biomedical data (Al-Sharqi et al., 2025). Given that the pathological progression of AD involves multidimensional and regionally distributed brain alterations, the superior sensitivity observed in the RF model likely reflects its enhanced capacity to detect such nonlinear patterns. From a clinical implementation perspective, algorithm selection may be tailored to specific application scenarios. For large-scale screening programs where high specificity is prioritized to reduce false positives, the Combined-LR model may represent a more appropriate option.

Conversely, for high-risk populations requiring precise diagnostic assessment, the Combined-RF model may be advantageous due to its higher sensitivity and lower likelihood of missed diagnoses. Compared with prior studies, the combined models proposed in this research demonstrate superior diagnostic performance. Chen et al. (2025) developed a multimodal radiomics model for AD diagnosis with a maximum test-set AUC of 0.899, while (Yin et al., 2024) reported an optimal test-set AUC of 0.961 based on hippocampal radiomic features derived from T1-weighted imaging. In contrast, the Combined-LR model in the present study achieved a test-set AUC of 0.989, and the Combined-RF model attained an AUC of 0.970, underscoring the enhanced diagnostic efficacy of the integrated multimodal framework.

Importantly, beyond performance improvement, this study further examined which multimodal feature combinations most accurately distinguished AD and linked these features to established pathological processes, including amyloid-β deposition, Tau neurofibrillary tangles, neuroinflammation, and synaptic dysfunction. By aligning predictive imaging signatures with known pathophysiological mechanisms, this approach not only strengthens biological interpretability but also provides a framework for validating existing hypotheses and potentially generating new mechanistic insights into AD progression.

### 4.4 Clinical significance of SHAP interpretability analysis

In this study, SHAP analysis was employed to mitigate the inherent “black-box” limitations of machine learning models by providing transparent and clinically coherent explanations for diagnostic predictions. The SHAP results not only quantified the individual contribution of each radiomic feature to the final classification outcome but, more importantly, demonstrated that the most influential diagnostic features were highly consistent with established pathophysiological mechanisms of AD.

The analysis demonstrated that texture features derived from the parietal lobe, particularly original_glrlm_ShortRunHighGrayLevelEmphasis_PL_3D-T1WI (Short-Run High Gray-Level Emphasis in the parietal lobe on the 3D-T1WI sequence) and original_glcm_Imc1_PL_DWI (Information Measure of Correlation 1 in the parietal lobe on the DWI sequence), played a critical positive role in driving the model's prediction toward AD. An elevated value of the former feature suggests reduced continuity of high gray-level regions within the image (corresponding to gray matter), which may reflect AD-related pathological alterations, such as neuronal loss in the parietal cortex leading to blurring of the gray–white matter boundary. Similarly, an increase in the latter feature indicates greater informational complexity in gray-level distribution, implying increased microstructural heterogeneity of brain tissue. This heterogeneity may be attributable to disorganized neuronal architecture and expansion of extracellular spaces in AD (Ahulló-Fuster et al., 2022). In addition, a shape feature extracted from the posterior cingulate cortex (PCC) on the DWI sequence, original_shape_MajorAxisLength_PCC_DWI, was identified as an important contributor. A reduction in this metric may reflect PCC volume atrophy, which is positively associated with the severity of memory impairment in patients with AD. Extensive neuroimaging evidence has established that the parietal lobe, particularly the precuneus and posterior cingulate cortex, represents one of the earliest and most vulnerable regions affected by AD pathology, including amyloid-β deposition and cerebral hypometabolism (Ahulló-Fuster et al., 2022; Jacobs et al., 2012). The fact that the proposed model autonomously prioritized microstructural abnormalities within the parietal lobe further supports the biological plausibility and neuropathological consistency of its predictive mechanisms.

Furthermore, the individualized interpretability provided by SHAP, as illustrated through decision plots, enables the generation of a personalized “imaging-based diagnostic rationale report” for each subject. Through this visualization, clinicians can clearly identify which specific imaging feature abnormalities, and in which brain regions, contributed to the model's final classification outcome. This personalized and visually transparent explanation of the decision-making process substantially enhances clinicians' confidence in AI-generated diagnostic results. By making the underlying reasoning explicit, the model evolves from a purely predictive instrument into an interactive, transparent, and trustworthy clinical decision-support system.

### 4.5 Addressing overfitting, generalizability, and methodological rigor

The authors acknowledge that the initial feature-to-sample ratio (856 features vs. 110 subjects) may raise concerns regarding potential overfitting. However, our rigorous three-step feature selection pipeline reduced features to 16 core variables using training-set-only procedures with strict 5-fold cross-validation. The consistent performance observed between training and independent test sets—with the Combined-LR model achieving comparable AUCs of 0.994 and 0.989, respectively—provides strong evidence that the model has not been substantially overfitted. Nonetheless, future multi-center studies with larger sample sizes would further validate the stability of these selected radiomic features.

Additionally, the single-center retrospective design, while ensuring homogeneity of imaging acquisition and clinical evaluation protocols, inevitably limits the generalizability of the findings to broader clinical populations. The model's performance across different MRI scanner vendors, field strengths, and acquisition protocols remains to be validated. However, we note that the selected radiomic features are derived from standardized feature definitions (PyRadiomics) and are not tied to scanner-specific parameters, suggesting potential for cross-site transferability. Furthermore, the consistent identification of parietal lobe texture features aligns well with established neuropathological knowledge, providing biological credibility that may enhance generalizability beyond statistical properties alone. Future external validation using multi-center datasets is essential to confirm the model's robustness across diverse clinical settings.

### 4.6 Relation to advanced imaging and molecular biomarkers

Although the present study utilized conventional MRI sequences (3D-T1WI, DWI, and T2WI), which are widely available, cost-effective, and routinely acquired in clinical settings, integration of advanced imaging modalities holds promise for augmenting diagnostic performance and biological depth. Techniques such as arterial spin labeling (ASL) perfusion imaging can capture cerebral blood flow alterations, potentially detecting hypoperfusion patterns in the posterior cingulate cortex and parietal regions that may precede structural atrophy (Alsop et al., 2015). Susceptibility-weighted imaging (SWI) and quantitative susceptibility mapping (QSM) can detect iron deposition, which has been increasingly recognized as a contributor to oxidative stress and neurodegeneration in AD (Ayton et al., 2017). Furthermore, amyloid PET and tau PET provide direct molecular visualization of the core pathological hallmarks of AD, namely Aβ plaques and neurofibrillary tangles (Jack et al., 2018). However, PET imaging is limited by high cost, radiation exposure, and restricted accessibility, particularly in primary care settings where early screening is most needed (Marcus et al., 2014). Similarly, cerebrospinal fluid (CSF) biomarkers, including Aβ42, p-tau, and t-tau, offer high diagnostic accuracy but require invasive lumbar puncture, limiting patient acceptance and routine monitoring (Zetterberg et al., 2014). The major advantage of our MRI-based radiomics approach lies in its non-invasiveness, low cost, and ability to extract pathophysiologically relevant information from standard clinical scans—features that are conducive to large-scale screening and longitudinal monitoring. Future work will explore integration of these advanced modalities as they become more accessible, aiming to build a multi-omics, multi-modal diagnostic framework that combines the complementary strengths of different techniques.

### 4.7 Considerations on deep learning and alternative approaches

While deep learning and transformer-based architectures have demonstrated remarkable performance in image classification tasks, their application in the present context presents several challenges. Such models require substantially larger sample sizes than were available in this study to achieve stable training and avoid overfitting, and their inherent opacity would compromise the interpretability objective central to our work. The SHAP-enhanced traditional machine learning approach, by contrast, offers transparent attribution of diagnostic decisions to specific imaging features, which is crucial for clinician trust and adoption. Nevertheless, as multi-center data become increasingly available and explainable deep learning techniques mature, future studies may explore hybrid approaches that combine the representational power of deep learning with interpretability frameworks to further advance AD diagnostic capabilities.

### 4.8 Limitations regarding the absence of an MCI group

The binary classification framework (AD vs. HC), while providing clear proof-of-concept for the multimodal radiomics approach, does not address the clinically most challenging diagnostic scenario: distinguishing MCI from normal aging or identifying which MCI patients will progress to AD. MCI represents a heterogeneous transitional state, and subtle pathological alterations at this stage may not be fully captured by the same feature set optimized for AD-HC discrimination. The texture features identified from the parietal lobe, while strongly associated with established AD pathology, may exhibit more subtle effect sizes or different directionality in MCI populations, potentially requiring refined thresholding or additional features (such as longitudinal trajectory metrics) to achieve comparable diagnostic performance. Furthermore, the absence of an MCI group precludes evaluation of whether our model can identify prodromal AD, which remains the ultimate clinical goal. Future prospective studies will incorporate MCI patients with longitudinal follow-up to assess the model's utility in predicting progression from MCI to AD—a more clinically actionable application that would allow timely therapeutic intervention. We hypothesize that the multimodal framework, particularly DWI-derived microstructural features, may be especially valuable in this early phase, given their sensitivity to subtle microstructural changes before overt atrophy develops.

### 4.9 Study limitations and future prospects

Despite the encouraging findings, several limitations should be acknowledged. First, this study was conducted at a single center using a retrospective design and a relatively modest sample size, which may introduce potential selection bias. Consequently, the generalizability and stability of the proposed model require further validation in larger, multi-center prospective cohorts. Second, the imaging protocol was limited to three conventional MRI sequences. Future investigations should consider incorporating advanced imaging modalities, such as ASL perfusion imaging, SWI, and amyloid PET. The integration of these techniques would allow the capture of additional pathological dimensions of AD, including cerebral hemodynamics, iron deposition, and amyloid burden. Such expansion would facilitate the development of a more comprehensive “imaging–clinical–molecular” multimodal diagnostic framework, thereby further enhancing diagnostic precision and biological interpretability. Third, the absence of an MCI group limits the evaluation of the model's utility in the most clinically relevant diagnostic gray zone. Fourth, manual ROI adjustments were performed without formal inter-observer reliability metrics, which could affect reproducibility. Finally, the lack of external validation across different scanners and institutions remains a key limitation.

Looking forward, our future research will focus on several key directions. First, we plan to establish multi-center collaborations to expand the sample size and acquire longitudinal follow-up data. This will enable external validation of the proposed model and further enhance its robustness, generalizability, and predictive performance across diverse populations and imaging platforms. Second, we aim to incorporate non-imaging biomarkers, including CSF indices and genetic data, and to explore advanced strategies for integrating multi-source heterogeneous data. By developing a unified multimodal fusion framework, we seek to construct a more comprehensive, accurate, and interpretable early diagnostic tool for AD, thereby accelerating the clinical implementation of artificial intelligence technologies in the management of neurodegenerative disorders. Third, we intend to conduct prospective longitudinal cohort studies to dynamically monitor changes in homocysteine and triglyceride levels from midlife to late adulthood. Such investigations will help clarify their temporal sequence and potential causal relationship with AD onset and progression. Finally, we will undertake mechanistic studies using AD animal models to examine the effects of targeted interventions on homocysteine and triglyceride levels. By evaluating their impact on amyloid-β deposition, Tau pathology, neuroinflammation, and cognitive function, we aim to directly verify potential synergistic interactions and elucidate the underlying biological mechanisms linking these metabolic factors to AD pathogenesis.

## 5 Conclusion

This study developed an interpretable machine learning framework based on multimodal MRI-derived radiomic features to support the auxiliary diagnosis of AD. The findings demonstrated that the integrated model incorporating 3D-T1WI, DWI, and T2-weighted sequences achieved significantly superior diagnostic performance compared with models constructed from any single imaging sequence. Among the evaluated algorithms, the logistic regression–based combined model (combined-LR) exhibited the strongest overall performance on the independent test set, attaining an AUC of 0.989. The key radiomic features identified through systematic feature selection, together with clinically relevant variables such as homocysteine and triglyceride levels determined through univariate and multivariate analyses, demonstrated substantial diagnostic relevance and clinical applicability. Moreover, the incorporation of SHAP analysis enhanced the interpretability of the predictive model by clarifying its internal decision-making logic. The results revealed that texture-related features of the parietal lobe served as the principal imaging biomarkers differentiating patients with AD from cognitively normal controls. Notably, this observation is highly consistent with established neuropathological and pathophysiological evidence regarding the progression of AD.

In summary, the integration of multimodal MRI-based radiomics with interpretable machine learning enables the development of highly efficient and accurate diagnostic models for AD while simultaneously offering transparent and clinically meaningful explanations for model decision-making. This combined framework establishes a robust theoretical and practical foundation for advancing the clinical translation and implementation of AI-assisted tools in the early detection, precise diagnosis, and individualized management of AD. Importantly, this approach signifies a paradigm shift in diagnostic strategy, transitioning from reliance on single-modality, subjective, and macro-level morphological assessments to a comprehensive, multiparametric, and objective systems-level analysis that integrates both macroscopic structural alterations and microscopic functional changes. From a practical perspective, a major advantage of this methodology lies in its capacity to support AD diagnosis through entirely non-invasive procedures. It can be implemented using standard MRI equipment commonly available in primary care hospitals, without requiring contrast agents, blood sampling, or CSF collection. Consequently, this approach reduces patient testing costs and procedural burden while simultaneously providing clinicians with rapid diagnostic outputs and interpretable supporting evidence to inform clinical decision-making.

## Data Availability

The raw data supporting the conclusions of this article will be made available by the authors, without undue reservation.
